# 3-(2-Methyl­benzyl­idene)-2,3-dihydro-1,5-benzothia­zepin-4(5*H*)-one

**DOI:** 10.1107/S1600536810052888

**Published:** 2011-01-08

**Authors:** D. Sridevi, Sundari Bhaskaran, G. Usha, G. Murugan, M. Bakthadoss

**Affiliations:** a Department of Physics, Queen Mary’s College(A), Chennai-4, Tamilnadu, India; b Department of Organic Chemistry, University of Madras, Guindy Campus, Chennai-25, India

## Abstract

In the crystal structure of the title compound, C_17_H_15_NOS, the mol­ecules form centrosymmetric dimers through pairs of N—H⋯O hydrogen bonds. The seven-membered ring adopts a distorted half-chair conformation.

## Related literature

Dibenzo[*c*,*e*]thiepine derivatives exhibit chiroptical properties (Tomascovic *et al.*, 2000 ▶) and dibenzo[*b*,*e*]thiepin-5,5-dioxide derivatives possess anti­histaminic and anti­allergenic activities (Rajsner *et al.*, 1971 ▶) while benzene thiepine derivatives have been identified as effective anti­histaminic compounds (Metys *et al.*, 1965 ▶).
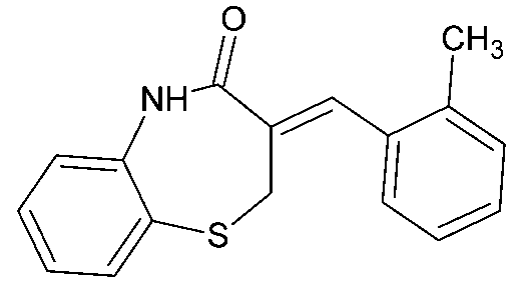


## Experimental

### 

#### Crystal data

C_17_H_15_NOS
*M* *_r_* = 281.36
Monoclinic, 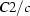

*a* = 19.1192 (5) Å
*b* = 13.0049 (3) Å
*c* = 14.8903 (4) Å
β = 128.591 (1)°
*V* = 2893.84 (13) Å^3^
*Z* = 8
Mo *K*α radiation
μ = 0.22 mm^−1^
*T* = 293 K
0.22 × 0.18 × 0.18 mm

#### Data collection

Bruker Kappa APEXII CCD diffractometer
13879 measured reflections
3560 independent reflections
2707 reflections with *I* > 2σ(*I*)
*R*_int_ = 0.024

#### Refinement

*R*[*F* ^2^ > 2σ(*F* ^2^)] = 0.044
*wR*(*F* ^2^) = 0.202
*S* = 0.83
3560 reflections
181 parameters
H-atom parameters constrained
Δρ_max_ = 0.27 e Å^−3^
Δρ_min_ = −0.30 e Å^−3^

### 

Data collection: *APEX2* (Bruker, 2004 ▶); cell refinement: *SAINT* (Bruker, 2004 ▶); data reduction: *SAINT* and *XPREP* (Bruker, 2004 ▶); program(s) used to solve structure: *SHELXS97* (Sheldrick, 2008 ▶); program(s) used to refine structure: *SHELXL97* (Sheldrick, 2008 ▶); molecular graphics: *ORTEP-3 for Windows* (Farrugia, 1997 ▶); software used to prepare material for publication: *SHELXL97* and *PLATON* (Spek, 2009 ▶).

## Supplementary Material

Crystal structure: contains datablocks I, global. DOI: 10.1107/S1600536810052888/bt5422sup1.cif

Structure factors: contains datablocks I. DOI: 10.1107/S1600536810052888/bt5422Isup2.hkl

Additional supplementary materials:  crystallographic information; 3D view; checkCIF report

## Figures and Tables

**Table 1:** Hydrogen-bond geometry (Å, °)

*D*—H⋯*A*	*D*—H	H⋯*A*	*D*⋯*A*	*D*—H⋯*A*
N—H0⋯O^i^	0.86	2.01	2.8705 (18)	177

Symmetry code: (i) 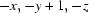.

**Fig. 1.**
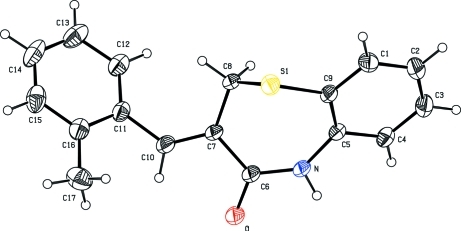
Perspective view of the title compound with 30% probability ellipsoids.

**Fig. 2.**
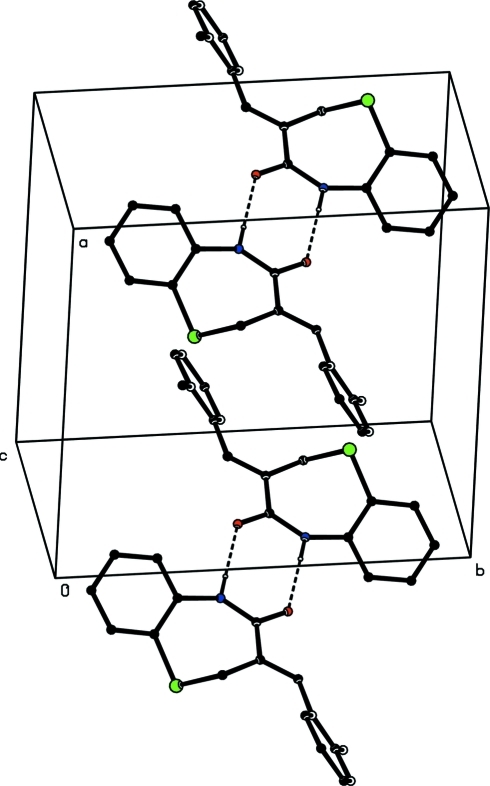
Partial packing diagram, viewed along the c axis.

## supplementary crystallographic information

### Comment

The title compound is used as an intermediate for the synthesis of dosulepin, which is an antidepressant of the tricyclic family. Dosulepin prevents reabsorbing of serotonin and noradrenaline in the brain, helps to prolong the mood lightening effect of any released noradrenaline and serotonin, thus relieving depression. The dibenzo[c,e]thiepine derivatives exhibit chiroptical properties (Tomascovic *et al.*, 2000). Dibenzo[b,e]thiepin-5,5-dioxide derivatives  possess antihistaminic and antiallergenic activities (Rajsner *et al.*, 1971). Benzene thiepine derivatives are identified as a new type of effective antihistaminic compounds (Metys *et al.*, 1965). Considering the wide range of biological activities of the thiepine derivatives, we determined the crystal structure  of the title compound. The seven membered thiepin ring adopts a distorted half-chair conformation.  The molecules form centrosymmetric dimers through N—H···O hydrogen bonds.

### Experimental

A mixture of(*Z*)-methyl 2-(bromomethyl)-3-*o*-tolylacrylate (2 mmol) and *o*-aminothiophenol(2 mmol) in the presence of potassium *tert*-butoxide (2.4 mmol) in dry THF (10 ml) was stirred at room temperature for 1 h. After the completion of the reaction as indicated by TLC, the reaction mixture was concentrated and the resulting crude mass was diluted with water (20 ml) and extracted with ethyl acetate (3 x 20ml). The organic layer was washed with brine (2 x 20ml) and dried over anhydrous sodium sulfate. The organic layer was concentrated, which provided a crude mass (*Z*)-methyl 2-((2-aminophenylthio)methyl)-3-*o*-tolylacrylate. The crude product was treated with a catalytic amount of *p*-toluene sulphonic acid (0.4 mmol), in p-xylene (10 ml), under reflux conditions for 12 h. After the completion of the reaction as indicated by TLC, the reaction mixture was concentrated under reduced pressure and worked up as mentioned previously, which successfully provide the crude final product. The final product was purified by column chromatography on silica gel to afford the  title compound in good yield (67%).

### Refinement

H atoms were positioned geometrically and were treated as riding on their parent atoms, with C—H distances of 0.93–0.97Å, an N—H distance of 0.86Å and *U*_iso_(H)=1.2*U*_eq_(N, C).

### Crystal data

**Table:**

C_17_H_15_NOS	*F*(000) = 1184
*M**_r_* = 281.36	*D*_x_ = 1.292 Mg m^−^^3^
Monoclinic, *C*2/*c*	Mo *K*α radiation, λ = 0.71073 Å
*a* = 19.1192 (5) Å	Cell parameters from 3560 reflections
*b* = 13.0049 (3) Å	θ = 2–28°
*c* = 14.8903 (4) Å	µ = 0.22 mm^−^^1^
β = 128.591 (1)°	*T* = 293 K
*V* = 2893.84 (13) Å^3^	Block, colourless
*Z* = 8	0.22 × 0.18 × 0.18 mm

### Data collection

**Table:**

Bruker Kappa APEXII CCD diffractometer	2707 reflections with *I* > 2σ(*I*)
Radiation source: fine-focus sealed tube	*R*_int_ = 0.024
graphite	θ_max_ = 28.4°, θ_min_ = 2.1°
ω and φ scans	*h* = −25→20
13879 measured reflections	*k* = −15→16
3560 independent reflections	*l* = −15→19

### Refinement

**Table:**

Refinement on *F*^2^	Primary atom site location: structure-invariant direct methods
Least-squares matrix: full	Secondary atom site location: difference Fourier map
*R*[*F*^2^ > 2σ(*F*^2^)] = 0.044	Hydrogen site location: inferred from neighbouring sites
*wR*(*F*^2^) = 0.202	H-atom parameters constrained
*S* = 0.83	*w* = 1/[σ^2^(*F*_o_^2^) + (0.2*P*)^2^] where *P* = (*F*_o_^2^ + 2*F*_c_^2^)/3
3560 reflections	(Δ/σ)_max_ < 0.001
181 parameters	Δρ_max_ = 0.27 e Å^−^^3^
0 restraints	Δρ_min_ = −0.30 e Å^−^^3^
0 constraints	

### Special details

**Table:**

Geometry. All e.s.d.'s (except the e.s.d. in the dihedral angle between two l.s. planes) are estimated using the full covariance matrix. The cell e.s.d.'s are taken into account individually in the estimation of e.s.d.'s in distances, angles and torsion angles; correlations between e.s.d.'s in cell parameters are only used when they are defined by crystal symmetry. An approximate (isotropic) treatment of cell e.s.d.'s is used for estimating e.s.d.'s involving l.s. planes.
Refinement. Refinement of *F*^2^ against ALL reflections. The weighted *R*-factor *wR* and goodness of fit *S* are based on *F*^2^, conventional *R*-factors *R* are based on *F*, with *F* set to zero for negative *F*^2^. The threshold expression of *F*^2^ > σ(*F*^2^) is used only for calculating *R*-factors(gt) *etc*. and is not relevant to the choice of reflections for refinement. *R*-factors based on *F*^2^ are statistically about twice as large as those based on *F*, and *R*- factors based on ALL data will be even larger.

### Fractional atomic coordinates and isotropic or equivalent isotropic displacement parameters (Å^2^)

**Table:**

	*x*	*y*	*z*	*U*_iso_*/*U*_eq_	
C1	0.07250 (13)	0.88179 (15)	0.21097 (16)	0.0540 (5)	
H1	0.1146	0.9233	0.2730	0.065*	
C2	−0.00643 (15)	0.92462 (17)	0.11794 (18)	0.0623 (5)	
H2	−0.0169	0.9947	0.1164	0.075*	
C3	−0.06956 (12)	0.86298 (15)	0.02748 (16)	0.0557 (5)	
H3	−0.1233	0.8912	−0.0351	0.067*	
C4	−0.05362 (11)	0.75966 (14)	0.02896 (14)	0.0448 (4)	
H4	−0.0970	0.7185	−0.0326	0.054*	
C5	0.02685 (10)	0.71567 (12)	0.12171 (12)	0.0365 (4)	
C6	0.08537 (12)	0.52859 (14)	0.17690 (13)	0.0485 (4)	
C7	0.14280 (11)	0.52918 (13)	0.30555 (12)	0.0424 (4)	
C8	0.15581 (12)	0.62540 (13)	0.36935 (13)	0.0478 (4)	
H8A	0.1988	0.6123	0.4512	0.057*	
H8B	0.0995	0.6441	0.3523	0.057*	
C9	0.09042 (10)	0.77806 (13)	0.21391 (13)	0.0398 (4)	
C10	0.18328 (12)	0.44083 (13)	0.35720 (14)	0.0477 (4)	
H10	0.1742	0.3876	0.3092	0.057*	
C11	0.24053 (11)	0.41648 (13)	0.48045 (14)	0.0449 (4)	
C12	0.21413 (14)	0.43985 (16)	0.54648 (16)	0.0573 (5)	
H12	0.1612	0.4759	0.5135	0.069*	
C13	0.26536 (17)	0.4103 (2)	0.66057 (18)	0.0723 (7)	
H13	0.2472	0.4269	0.7040	0.087*	
C14	0.34253 (16)	0.35663 (19)	0.70886 (18)	0.0750 (7)	
H14	0.3770	0.3360	0.7853	0.090*	
C15	0.36932 (13)	0.33310 (17)	0.64501 (18)	0.0676 (6)	
H15	0.4222	0.2966	0.6792	0.081*	
C16	0.31970 (11)	0.36225 (14)	0.52991 (15)	0.0504 (4)	
C17	0.35129 (16)	0.33506 (19)	0.4639 (2)	0.0722 (6)	
H17A	0.4068	0.2983	0.5126	0.108*	
H17B	0.3601	0.3967	0.4366	0.108*	
H17C	0.3074	0.2926	0.3997	0.108*	
N	0.03793 (9)	0.61072 (12)	0.10949 (10)	0.0450 (4)	
H0	0.0047	0.5939	0.0381	0.054*	
O	0.07844 (12)	0.44836 (11)	0.12814 (11)	0.0766 (5)	
S1	0.19459 (3)	0.73150 (4)	0.33323 (3)	0.0503 (2)	

### Atomic displacement parameters (Å^2^)

**Table:**

	*U*^11^	*U*^22^	*U*^33^	*U*^12^	*U*^13^	*U*^23^
C1	0.0582 (10)	0.0419 (10)	0.0457 (9)	−0.0007 (8)	0.0246 (8)	−0.0069 (7)
C2	0.0737 (12)	0.0426 (10)	0.0558 (11)	0.0134 (8)	0.0332 (10)	0.0055 (8)
C3	0.0507 (9)	0.0560 (12)	0.0456 (10)	0.0109 (8)	0.0228 (8)	0.0110 (8)
C4	0.0400 (8)	0.0503 (10)	0.0332 (8)	−0.0013 (6)	0.0174 (7)	0.0027 (6)
C5	0.0373 (7)	0.0405 (8)	0.0284 (7)	−0.0002 (6)	0.0190 (6)	0.0005 (6)
C6	0.0570 (9)	0.0436 (10)	0.0281 (7)	0.0032 (7)	0.0183 (7)	−0.0047 (6)
C7	0.0501 (8)	0.0414 (9)	0.0262 (7)	0.0001 (7)	0.0192 (6)	−0.0041 (6)
C8	0.0600 (9)	0.0448 (10)	0.0258 (7)	0.0080 (7)	0.0205 (7)	−0.0024 (6)
C9	0.0391 (7)	0.0424 (9)	0.0305 (7)	0.0003 (6)	0.0180 (6)	0.0004 (6)
C10	0.0579 (9)	0.0426 (10)	0.0323 (8)	−0.0020 (7)	0.0231 (8)	−0.0030 (7)
C11	0.0497 (8)	0.0391 (9)	0.0345 (8)	−0.0061 (6)	0.0207 (7)	0.0027 (6)
C12	0.0610 (10)	0.0628 (13)	0.0444 (10)	−0.0029 (9)	0.0310 (9)	0.0050 (8)
C13	0.0867 (16)	0.0852 (17)	0.0483 (11)	−0.0180 (12)	0.0438 (12)	0.0024 (11)
C14	0.0811 (14)	0.0757 (16)	0.0366 (9)	−0.0188 (12)	0.0211 (10)	0.0107 (9)
C15	0.0500 (9)	0.0583 (13)	0.0547 (12)	−0.0067 (8)	0.0131 (9)	0.0079 (9)
C16	0.0489 (8)	0.0429 (10)	0.0442 (9)	−0.0116 (7)	0.0217 (8)	−0.0030 (7)
C17	0.0664 (12)	0.0654 (14)	0.0809 (16)	−0.0040 (10)	0.0441 (12)	−0.0149 (11)
N	0.0499 (7)	0.0433 (8)	0.0229 (6)	0.0019 (6)	0.0135 (6)	−0.0046 (5)
O	0.1042 (12)	0.0492 (9)	0.0302 (6)	0.0191 (8)	0.0193 (7)	−0.0072 (5)
S1	0.0394 (3)	0.0464 (3)	0.0371 (3)	−0.00100 (15)	0.0101 (2)	−0.00664 (16)

### Geometric parameters (Å, °)

**Table:**

C1—C2	1.378 (3)	C9—S1	1.7538 (16)
C1—C9	1.386 (2)	C10—C11	1.470 (2)
C1—H1	0.9300	C10—H10	0.9300
C2—C3	1.373 (3)	C11—C16	1.393 (3)
C2—H2	0.9300	C11—C12	1.390 (3)
C3—C4	1.375 (3)	C12—C13	1.385 (3)
C3—H3	0.9300	C12—H12	0.9300
C4—C5	1.397 (2)	C13—C14	1.363 (3)
C4—H4	0.9300	C13—H13	0.9300
C5—C9	1.392 (2)	C14—C15	1.367 (4)
C5—N	1.410 (2)	C14—H14	0.9300
C6—O	1.230 (2)	C15—C16	1.397 (3)
C6—N	1.355 (2)	C15—H15	0.9300
C6—C7	1.501 (2)	C16—C17	1.482 (3)
C7—C10	1.330 (2)	C17—H17A	0.9600
C7—C8	1.495 (2)	C17—H17B	0.9600
C8—S1	1.8001 (19)	C17—H17C	0.9600
C8—H8A	0.9700	N—H0	0.8600
C8—H8B	0.9700		
			
C2—C1—C9	121.29 (17)	C7—C10—H10	115.8
C2—C1—H1	119.4	C11—C10—H10	115.8
C9—C1—H1	119.4	C16—C11—C12	119.40 (16)
C3—C2—C1	119.44 (19)	C16—C11—C10	119.11 (17)
C3—C2—H2	120.3	C12—C11—C10	121.33 (17)
C1—C2—H2	120.3	C13—C12—C11	121.1 (2)
C2—C3—C4	120.29 (17)	C13—C12—H12	119.5
C2—C3—H3	119.9	C11—C12—H12	119.5
C4—C3—H3	119.9	C14—C13—C12	119.6 (2)
C3—C4—C5	120.83 (16)	C14—C13—H13	120.2
C3—C4—H4	119.6	C12—C13—H13	120.2
C5—C4—H4	119.6	C15—C14—C13	119.99 (19)
C9—C5—C4	118.81 (15)	C15—C14—H14	120.0
C9—C5—N	125.61 (14)	C13—C14—H14	120.0
C4—C5—N	115.48 (14)	C14—C15—C16	122.0 (2)
O—C6—N	117.10 (14)	C14—C15—H15	119.0
O—C6—C7	118.78 (15)	C16—C15—H15	119.0
N—C6—C7	124.09 (14)	C11—C16—C15	117.93 (19)
C10—C7—C8	123.34 (14)	C11—C16—C17	121.88 (18)
C10—C7—C6	115.63 (14)	C15—C16—C17	120.2 (2)
C8—C7—C6	120.97 (14)	C16—C17—H17A	109.5
C7—C8—S1	112.83 (12)	C16—C17—H17B	109.5
C7—C8—H8A	109.0	H17A—C17—H17B	109.5
S1—C8—H8A	109.0	C16—C17—H17C	109.5
C7—C8—H8B	109.0	H17A—C17—H17C	109.5
S1—C8—H8B	109.0	H17B—C17—H17C	109.5
H8A—C8—H8B	107.8	C6—N—C5	138.81 (13)
C1—C9—C5	119.31 (15)	C6—N—H0	110.6
C1—C9—S1	118.05 (13)	C5—N—H0	110.6
C5—C9—S1	122.61 (13)	C9—S1—C8	98.53 (8)
C7—C10—C11	128.30 (16)		
			
C9—C1—C2—C3	1.6 (3)	C7—C10—C11—C12	49.1 (3)
C1—C2—C3—C4	−0.9 (3)	C16—C11—C12—C13	0.1 (3)
C2—C3—C4—C5	−0.3 (3)	C10—C11—C12—C13	175.39 (19)
C3—C4—C5—C9	1.0 (2)	C11—C12—C13—C14	−0.6 (3)
C3—C4—C5—N	−175.59 (16)	C12—C13—C14—C15	0.5 (4)
O—C6—C7—C10	0.9 (3)	C13—C14—C15—C16	−0.1 (3)
N—C6—C7—C10	178.94 (17)	C12—C11—C16—C15	0.3 (3)
O—C6—C7—C8	178.10 (19)	C10—C11—C16—C15	−175.08 (17)
N—C6—C7—C8	−3.8 (3)	C12—C11—C16—C17	179.97 (18)
C10—C7—C8—S1	123.26 (16)	C10—C11—C16—C17	4.6 (3)
C6—C7—C8—S1	−53.7 (2)	C14—C15—C16—C11	−0.3 (3)
C2—C1—C9—C5	−0.9 (3)	C14—C15—C16—C17	−180.0 (2)
C2—C1—C9—S1	177.38 (15)	O—C6—N—C5	−175.66 (19)
C4—C5—C9—C1	−0.4 (2)	C7—C6—N—C5	6.3 (3)
N—C5—C9—C1	175.84 (16)	C9—C5—N—C6	26.7 (3)
C4—C5—C9—S1	−178.59 (12)	C4—C5—N—C6	−157.0 (2)
N—C5—C9—S1	−2.4 (2)	C1—C9—S1—C8	128.73 (15)
C8—C7—C10—C11	5.3 (3)	C5—C9—S1—C8	−53.03 (15)
C6—C7—C10—C11	−177.56 (18)	C7—C8—S1—C9	87.11 (13)
C7—C10—C11—C16	−135.7 (2)		

### Hydrogen-bond geometry (Å, °)

**Table:**

*D*—H···*A*	*D*—H	H···*A*	*D*···*A*	*D*—H···*A*
N—H0···O^i^	0.86	2.01	2.8705 (18)	177

Symmetry codes: (i) −*x*, −*y*+1, −*z*.
